# A Greater Need for Professional Dignity

**Published:** 1895-06

**Authors:** 


### Editorial.

### A GREATER NEED FOR PROFESSIONAL DIGNITY.

The distinctions drawn in the various positions in life may be of an arbitrary character, yet they are based on experience. That they are misused is true, and individuals are set off into classes from which it is difficult, if not impossible, to rise, as in the older civilizations. This is the extreme abuse of what was originally a natural and, without doubt, a serviceable division.

Training and association have largely to do with the positions occupied in newly-settled communities. The child of poverty and coarse associations may, by the force and upward tendency of education and its refining influence, reach a station in life in the New World not possible in the more conservative elements of the Old.

All this is well understood, but while education and refining environments may do much to raise to a higher class, it cannot obliterate the strain of inheritance in one generation. This remains and will show itself as plainly in the millionaire as in the lowly-born. It may require generations of wealth to produce that love of refinement so perceptible in lives of quiet, unselfish work, so frequently seen in what are termed the middle class families.

We are led to these prefatory remarks by the observation that the so-called dental profession is in many respects not what it ought to be when viewed as a whole. There are wide differences in individuals, some with a full appreciation of the importance and dignity of their calling, while there is another class who, while appreciating the work and the necessity of ethical culture to maintain its integrity, is still filled with the leaven of earlier surroundings and give to the work and its associative efforts a degree of unrefinement at once unpleasant and oftentimes shocking. There is still a lower class without the slightest regard for the higher life, and this will sacrifice ethics, the good opinions of their fellows, for temporary emoluments. These different classes shade oft into each other and have no clearly-defined limitations. While this is true of dentistry, it is by no means confined to it as a profession, but all, theology, medicine, law, and the continually developing newer divisions, have the same unpleasant combinations and perhaps to an equal extent as our own calling. It is not our purpose, however, to labor outside of our own vineyard.

The result of this commingling of the coarser with the more refined produces naturally inharmonious elements. We see it everywhere, and it is possible that there may be no immediate hope of improvement. It may certainly be laid down as a law of professional life that the individual who fails to aspire to the highest qualities attainable, in manners, language, and correct life, is unworthy to wear the professional mantle no matter how many degrees he may have earned from the schools.

The ill-bred man may not render himself specially obnoxious in his own office, but patients will mark a difference, and while for the sake of skill they may bear with unrefinement, it in the end tends not only to individual loss but casts a dark shadow on the profession.

It was through this evil, as much as a lack of thorough medical training, that led dentists of a former era to be regarded with contempt by medical men and with very little respect by the community at large. As this changed there has arisen a higher respect and a more intelligent appreciation of the service performed, and just in proportion as this refinement is increased will dentistry hold a more advanced position.

The evidence of this unrefined life is more to be found in journals devoted to the interest of trade as distinct from that felt for the profession. The criticism we make does not apply to the higher class of journals of the trade series; indeed, in several it has no place, but in some recent additions the violations of professional good breeding are painfully apparent. Witticisms, whether new or old, have no place in a dental journal, nor should doggerel rhymes and anecdotes from the newspapers relating to dentistry be recognized.

Professional life is a serious thing. There is no time in connection with it for the frivolities common in the jar and tumult of ordinary trade. These may have a proper place in the social whirl, but never in a journal devoted to the higher training of its readers.

The same criticism can be applied, and with equal force, to our associations. It is one of the burdens of editors that they are forced to eliminate all these labored witticisms and out-of-place remarks. If speakers would confine themselves to the subject, and stop when they have finished, there would be a higher standard very soon developed. The funny man has no place in meetings of a scientific character, for these gatherings mean an attempt to find truth, and there is nothing so inappropriate in a serious effort in this direction as the jester's wit, for it is always "much more easy to raise a laugh than to excite admiration by quick wisdom."

Editors of reports of dental societies should unsparingly cut out all irrelevant remarks. If this were faithfully done, dental societies would not be long burdened by such misplaced effusions.

Journals that persist in lowering the standard of dignified expression should be permitted to find their own level, and that will be among those who flourish behind the golden tooth attached to the iron rod, for they can have no place among the self-respecting readers of a dignified literature.

